# How I Manage Chronic Lymphocytic Leukemia

**DOI:** 10.3390/hematolrep15030047

**Published:** 2023-08-01

**Authors:** Patrice Nasnas, Claudio Cerchione, Gerardo Musuraca, Giovanni Martinelli, Alessandra Ferrajoli

**Affiliations:** Department of Leukemia, The University of Texas MD Anderson Cancer Center, Houston, TX 77030, USA

**Keywords:** CLL, targeted therapies, Bruton tyrosine kinase inhibitors, BCL2 inhibitor

## Abstract

Chronic lymphocytic leukemia (CLL), is a hematologic malignancy characterized by the uncontrolled proliferation of mature B lymphocytes. CLL is the most prevalent leukemia in Western countries. Its presentation can range from asymptomatic with the incidental finding of absolute lymphocytosis on a routine blood test, to symptomatic disease requiring immediate intervention. Prognosis of the disease is defined by the presence or absence of specific mutations such as TP53, chromosomal abnormalities such as del(17p), a type of IGHV mutational status, and elevation of B2M and LDH. Treatment of CLL in the United States and Europe has evolved over the recent years thanks to the development of targeted therapies. The standard of care has shifted from traditional chemoimmunotherapy approaches to targeted therapies including Bruton tyrosine kinase inhibitors (BTKis) and BCL2 inhibitors, administered either as monotherapy or in combination with CD20 monoclonal antibodies. Several clinical trials have also recently evaluated combinations of BTKi and venetoclax and showed the combination to be well tolerated and able to induce deep remissions. Targeted therapies have a good safety profile overall; however, they also have unique toxicities that are important to recognize. Diarrhea, fatigue, arthralgia, infections, cytopenias, bleeding, and cardiovascular toxicities (including atrial fibrillation, ventricular arrhythmias, and hypertension) are the adverse events (AEs) commonly associated with BTKis. Initiation of therapy with venetoclax requires close monitoring because of the risk for tumor lysis syndrome associated with this agent, particularly in patients with a high disease burden. Development of newer target therapies is ongoing and the therapeutic landscape in CLL is expanding rapidly.

## 1. Introduction and Epidemiology

Chronic lymphocytic leukemia (CLL) is a hematological malignancy characterized by the relentless accumulation of monoclonal mature B-lymphocytes in the peripheral blood, bone marrow, and lymphoid tissue. The SEER estimates that in 2023, approximately 18,740 individuals will be diagnosed with CLL in the United States and 4490 will die from the disease. Worldwide, there are approximately 191,000 case and 61,000 deaths per year attributed to CLL/SLL [1,2]. CLL is a disease of older individuals, the median age at diagnosis is 72 years, it has a male predominance with a male: female ratio of 2:1 and most of the patients are white (6.4 new cases per 100,000 for men vs. 3.4 for women, and 7.7 for non-Hispanic whites as the highest ratio for races) [3].

## 2. Diagnosis and Clinical Presentation

Diagnosing CLL requires the documented presence of ≥5 × 10^9^/L B lymphocytes in the peripheral blood, sustained for at least 3 months. Leukemia cells found on blood smear are small mature-appearing lymphocytes with a scant cytoplasm and a dense nucleus, lacking discernable nucleoli and partially aggregated chromatin. CLL cells tend to break during slide preparation and cell debris are easily identified and given the name of Gumprecht shadows or basket cells [2].

Confirmation of diagnosis requires performing flow cytometry analysis to identify the unique phenotype of the clonal population that is characterized by aberrant CD19-CD5 co-expression and surface immunoglobulin light chain restriction. In cases with a typical immunophenotype, a panel that includes CD19, CD5, CD20, CD23, and kappa and lambda light chain renders the diagnosis; however, larger panels are often used to further support the diagnosis and allow the differentiation with other CD5 positive lymphoproliferative disorders [4].

The presenting features of CLL may vary from asymptomatic patients found to have leukocytosis with lymphocytosis on a routine blood count, to other patients noticing painless enlargement of superficial lymph nodes. Overall, 5 to 10% of patients present with typical B symptoms of lymphoma that include one or more of the following: unintentional weight loss to more than 10% of their body weight within the previous 6 months, fevers for ≥2 weeks without evidence of infection, drenching night sweats, and fatigue. Occasionally, patients are diagnosed because of the development of symptoms related to autoimmune manifestations such as autoimmune hemolytic anemia or immune-mediated thrombocytopenia [2,5].

Small lymphocytic lymphoma (SLL) and CLL have identical pathologic and immunophenotypic features. The key difference is that in CLL, there is a significant number of abnormal lymphocytes found in circulation as well as involvement of bone marrow and lymphoid tissues, while in SLL, the cells occupy the same tissues, but without the leukemic appearance (WHO classification). Together, CLL and SLL are the most prevalent types of leukemia in Western countries, accounting for 25 to 35% of all leukemias in the United States and treatment approaches and supportive care for CLL and SLL is superimposable. In this educational paper, we will use the term CLL to define both diagnoses.

## 3. Staging and Prognostic Factors

Patients with CLL are staged according to the Rai or Binet systems with the Rai system used routinely in the US and the Binet system utilized in Europe. Both staging systems (Table 1A,B) are based on physical exam and laboratory parameters; they are simple, rapid to perform, and provide an assessment of the bulk of the disease and correlate with clinical outcomes [2,3,6]. Patients with SLL are staged according to the Lugano staging system of NHL that is a modification of the original Ann Arbor staging system [3].

Several biological features add important prognostic information that is independent from clinical staging at diagnosis. The prognostic markers that are widely available and frequently used in clinical practice include status of the immunoglobulin heavy chain variable region (IGHV) gene mutation, cytogenetic abnormalities by fluorescence in situ hybridization (FISH), CpG-stimulated karyotyping, cell surface markers expression, beta2microglobulin, LDH serum levels [7,8,9], and gene mutations by whole genome sequencing (summarized in Table 2).

## 4. Management and Treatment (Including US and European Perspectives)

A unique feature of CLL is that not all patients require treatment at the time of diagnosis. Patients without symptoms and Rai stage 0–II or Binet stages A and B are candidates for a period of “watch and wait” since the disease can have an indolent course and, furthermore, early intervention trials have not resulted in a survival advantage, in either earlier trials with chemotherapy or monoclonal antibody-based interventions, and, in more recent clinical studies with targeted therapies [11]. The International Workshop on Chronic Lymphocytic Leukemia (iwCLL) guidelines indicate that treatment is required in patients with “active disease” [12], with evidence of progressive marrow failure, as evident by worsening anemia or thrombocytopenia, massive or progressive symptomatic splenomegaly or lymphadenopathy, and progressive lymphocytosis with an increase of >50% over a 2-month period, or a lymphocyte doubling time (LDT) of <6 months. Cut off levels of Hb < 11 g/dL or platelet counts <100 × 10^9^/L are generally regarded as indications for treatment. In patients with plans for initiating treatment, patient age, performance status or fitness level, and the presence or absence of del(17p) or TP53 mutation and IGHV mutation status could help to guide treatment selection. Re-evaluation for TP53 mutation status and del(17p) by FISH, and IGHV mutation status if not previously established (important for selection of initial treatment if considering chemoimmunotherapy) are recommended prior to initiating subsequent treatments. CpG-stimulated karyotyping is also useful to identify high-risk patients, including patients who will receive targeted therapies [6,7].

Assessing response to treatment is an important element in the management of patients with CLL; however, stringent response criteria and bone marrow evaluations at the time of response assessment are not always followed in patients who are treated outside clinical trials. Evaluation of response includes assessment of resolution or worsening of symptoms, changes in lymphadenopathy extent as evident by clinical exam and/or radiographic measurements to determine the type of response (complete remission, partial remission, stable disease, or disease progression), and bone marrow evaluation if complete response is considered based on iwCLL criteria. The assessment of measurable residual disease (MRD) is usually integrated in the response assessment of patients enrolled in clinical trial and should be performed in all patients, especially in patients who are young and in patients with high-risk features. There are several techniques for assessing MRD, if they have undergone a critical evaluation and have become well standardized. Six-color flow cytometry (MRD flow), allele-specific oligonucleotide PCR, or high-throughput immunosequencing, such as by ClonoSEQ assay, are reliable and sensitive assays able to reach a sensitivity level equal to or less than one CLL cell in 10,000 leukocytes [3]. A widely used flow cytometry-based assay comprises a core panel of six markers (i.e., CD19, CD20, CD5, CD43, CD79b, and CD81) and with this assay, patients will be defined as having undetectable MRD (MRD-neg) remission if they have blood or marrow with less than one CLL cell per 10,000 leukocytes [7].

## 5. Treatment

For patients of all ages requiring treatment initiation in the US, participation in clinical trials is highly encouraged. Several academic and non-academic centers offer such an option for both treatment-naïve and previously treated patients. If clinical trial participation is not possible, the NCCCN guidelines offer expert advice in treatment selections and are regularly updated by a team of disease experts. Management and treatment practice in European countries often follows the guidelines published in 2020 by the ESMO and while in general similar to the NCCN guidelines, they differ based on European approval of certain agents [13]. Updates are expected for these guidelines in the near future given that additional agents (such as the covalent BTKi zanubrutinib) have received recent approval in several EU countries. Patients residing in Europe are encouraged to take part in clinical trials both in first-line and relapsed settings and several options are offered. For patients treated as part of a clinical trial and for patients treated as per the standard of care, improvement in quality of life and prolonging survival are the relevant goals of treatment. For young and fit patients, additional treatment endpoints such as achievement of minimal residual disease (MRD) status should be included when appropriate. Given the availability of several drugs with different mechanisms of action and toxicity profile, the choice of the treatment is dependent on both disease-related factors and patient factors. Evaluations to be completed prior to making a treatment recommendation comprises both del(17p)/TP53 evaluation to be conducted prior to any line of therapy and Ig heavy chain variable (IGHV) gene mutation status analysis if not known [14,15]. Patient factors that influence treatment include fitness, comorbidities (with a focus on novel drug-specific potential toxicity), ability to be monitored closely with laboratory evaluations and clinic visits, and, when possible, patient preference for continuous or fixed-duration treatment options.

Preferred initial treatments in the US for patients with and without del17p/TP53 abnormalities and regardless of age include targeted therapy with the second-generation BTKi acalabrutinib and zanubrutinib with or without anti-CD20 mAb or treatment with the BCL2 inhibitor venetoclax in combination with obinutuzumab.

**Figure 1:**
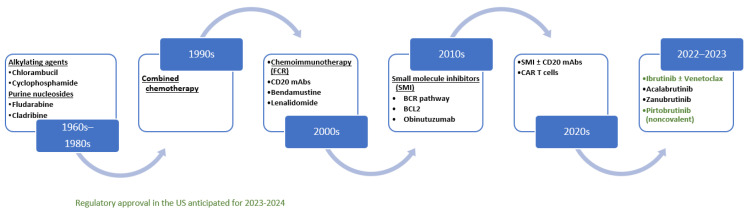
Summary of treatment evolution for CLL.

Treatment with ibrutinib as monotherapy or in combination with anti-CD mAbs is also recommended as category 1a given the availability of long-term efficacy data. Ibrutinib, acalabrutinib, and zanubrutinib bind to the ATP binding site of the BCR pathway covalently and in an irreversible manner, resulting in the reduction in downstream signaling impacting survival, proliferation, and interactions within the microenvironment and the endothelium. Ibrutinib was the first in class to be investigated and to be FDA approved for the treatment of patients with CLL. Ibrutinib received a breakthrough therapy designation for patients with CLL who carry del 17p/TP53 abnormalities in 2013. Ahn et al. reported a long-term observation of 34 patients who had CLL with TP53 alterations and were treated with ibrutinib as first-line therapy in the context of a phase 2 trial. After 6 years, the estimated percentage of patients with PFS and OS was 61% and 79%, respectively. Of the 12 patients who had disease progression while receiving ibrutinib, 4 had histologic transformation and 8 had progressive CLL. The data indicate that ibrutinib given as monotherapy has the ability to control high-risk, TP53 aberrant CLL over extended periods of time in some patients [16]. It should be noted, however, that genetic TP53 aberrations remain an unfavorable prognostic factor in the context of continuous BTK inhibitor monotherapy when compared to other factors. The final analysis of the RESONATE study showed that the presence of del(17p)/TP53 mutation or CK was not associated with inferior PFS outcomes to ibrutinib. A summary of treatment options is displayed in Figure 1.

European treatment recommendations for frontline therapy also depend on TP53 mutation or del(17p), IGHV mutational status, patient fitness, and comorbidities, and on the availability of certain agent in the country where treatment is given. Regardless of IGHV status and fitness, patients with CLL bearing TP53 mutations or del(17p) deletion should not receive chemoimmunotherapy and the preferred choice of treatment is for targeted therapies.

Ibrutinib monotherapy was approved for first-line therapy for all patients based on the results of the RESONATE-2 study that established the efficacy of ibrutinib monotherapy as first-line therapy in patients 65 years or older without del(17p). A long-term follow-up of the study has shown an OS rate of 78%, confirming the long-term value of first-line ibrutinib treatment, including for patients with high-risk disease features. An OS benefit for ibrutinib vs. chlorambucil has been established in analyses both with and without censoring for crossover, with HRs of 0.376 (95% CI, 0.180–0.786) and 0.450 (95% CI, 0.266–0.761), respectively. Ibrutinib given in combination with rituximab was more effective than FCR for patients 70 years or less without del(17p)/TP53 mutation, especially for those with unmutated IGHV, indicating that ibrutinib may also be an appropriate option for younger patients with IGHV unmutated CLL. Ibrutinib has been approved in Europe since 2014, with indication in both treatment-naïve and previously treated patients with CLL.

Acalabrutinib, a second-generation BTKi, was developed with the intention of improving the toxicity profile of BTKi by limiting the off-target effects. It was approved in the US in 2019. In a phase 1–2 study, 61 patients with relapsed CLL were treated with acalabrutinib at doses of 100–400 mg once daily in the dose-escalation (phase 1) portion of the study and 100 mg twice daily in the expansion (phase 2) portion, no dose-limiting toxic effects occurred during the dose-escalation portion of the study. An updated and expanded analysis of the study confirmed the efficacy, durability of response, and safety profile of acalabrutinib. Overall, 134 patients with relapsed/refractory CLL or SLL received acalabrutinib 100 mg twice daily for a median of 41 months [9,17]. Acalabrutinib was investigated as initial therapy in the phase 3 ELEVATE-TN trial. This study demonstrated that both acalabrutinib as monotherapy and the combination of acalabrutinib + obinutuzumab result in superior PFS versus chlorambucil + obinutuzumab in patients with previously untreated CLL. Both acalabrutinib-containing arms were associated with a PFS benefit in patients with unmutated IGHV as well as mutated IGHV compared to patients treated with chlorambucil + obinutuzumab. There was a trend towards improved OS in favor of the acalabrutinib-containing arms despite crossover for disease progression in the chlorambucil + obinutuzumab arm, though longer follow-up is needed to confirm any OS benefit. At 48-month follow-up, longer PFS (87% vs. 78%) was seen with acalabrutinib + obinutuzumab compared to acalabrutinib as monotherapy, although the study was not planned or powered to compare the PFS benefit between the two acalabrutinib arms. In the ELEVATE-TN study, the PFS benefit for acalabrutinib ± obinutuzumab was seen across all patient subgroups including those with del(17p) or TP53 mutation but only 14% of patients had del(17p) CLL. The 48-month PFS rates were 75% and 76%, respectively, for acalabrutinib + obinutuzumab and acalabrutinib monotherapy in patients with del(17p) and/or TP53 mutation.

Acalabrutinib has also been approved in Europe by the EMA since 2020, as a single agent in patients with CLL who have had exposure to previous treatment and combined with obinutuzumab for treatment-naïve patients [18].

Zanubrutinib is another second-generation BTKi and was developed to improve binding to BTK. Compared to acalabrutinib, it is less selective in its spectrum of kinases inhibition. Zanubrutinib was initially tested in a phase 1 study of various B cell malignancies. Additional data were gained from a phase 2 trial using zanubrutinib 160 mg twice daily in 91 Chinese patients with relapsed CLL. He study reported an ORR of 82–86% in patients with low- and high-risk CLL. While bleeding-associated AEs, including petechiae or contusions, were quite common (35%), atrial fibrillation (AF) was not observed. In the phase 3 SEQUOIA study, zanubrutinib resulted in higher ORR (95% vs. 85%) and statistically significant improvement in PFS compared to bendamustine and rituximab (BR) in patients with untreated CLL without del (17p)/TP53 mutation (HR 0.42; *p* < 0.0001) [19].

A head-to-head comparison between ibrutinib and zanubrutinib in patients with relapsed/refractory CLL was performed in the ALPINE phase 3 study. Patients enrolled in this study were treated with zanubrutinib or ibrutinib until intolerance or disease progression. The primary hypothesis was superiority of zanubrutinib compared to ibrutinib in terms of ORR, excluding those patients with PR with lymphocytosis. After a median follow-up of 15 months, a statistically showed a significantly higher, modified ORR of 78% for patients treated with zanubrutinib versus 63% for patients with ibrutinib was observed [19]. In Europe, zanubrutinib has been approved by the EMEA as single agent for CLL patients, regardless of previous treatment line number, since November 2022. Zanubrutinib has recently been approved by the FDA for the treatment of CLL (announced on 19 January 2023) [20].

Venetoclax is a BH3-mimetic compound that selectively antagonizes BCL-2 and induces apoptosis of CLL cells. Its efficacy as monotherapy has been described in patients with relapsed/refractory CLL, including those with del(17p). Venetoclax received initial approval in 2016 based on a phase 2 trial evaluating patients with relapsed/refractory disease with del(17p) [7]. The combination of venetoclax and rituximab was investigated in patients with relapsed CLL (MURANO trial) and showed that among patients with relapsed or refractory chronic lymphocytic leukemia, venetoclax plus rituximab resulted in significantly higher rates of progression-free survival than bendamustine plus rituximab, with a 2-year PFS of 84.9% as opposed to 36.3% [21].

The combination of venetoclax and obinutuzumab was compared to the combination of chlorambucil and venetoclax in a phase 3 randomized trial carried out by the German CLL study group (CLL14). The study investigated a fixed-duration treatment with venetoclax and obinutuzumab in patients with previously untreated CLL and coexisting conditions. In the CLL14 study, the PFS benefit for venetoclax + obinutuzumab were seen across all patient subgroups including a small number of with del(17p) or TP53 mutation (8% and 12% of patients, respectively). This regimen was established as an effective fixed-duration chemotherapy-free first-line treatment with significant improved PFS compared to traditional chlorambucil and obinutuzumab, at a median follow-up of 28 months in the CLL14 study; the 24-month PFS rate was 88% with FD venetoclax plus obinutuzumab [21]. This combination has been granted FDA approval for patients with CLL.

The combination of two targeted therapies, ibrutinib and venetoclax has been evaluated in a phase 2–3 clinical trial, CAPTIVATE study, that includes minimal residual disease (MRD)-guided treatment discontinuation following the completion of first-line ibrutinib plus venetoclax treatment. In the CAPTIVATE fixed-duration cohort, the 24-month PFS rate of 95%, together with results from the CAPTIVATE MRD cohort demonstrating 30-month PFS rates of ≥95% in placebo-randomized patients, with confirmed uMRD given MRD-guided treatment, supports the potential for durable, treatment-free remissions with fixed-duration ibrutinib plus venetoclax. Patients in the CAPTIVATE study are a young, fit population (aged ≤ 70 years with creatinine clearance (CrCL) ≥ 60 mL/min) typical of patients who may be considered eligible for treatment with FCR. Consequently, CAPTIVATE results may not be generalizable to the overall population of patients with CLL who tend to be older and have comorbidities [9,22]. The randomized phase 3 GLOW study evaluated the combination of ibrutinib and venetoclax in a population of unfit/elderly patients (aged ≥ 65 years or 18–64 years with a cumulative illness rating scale score > 6 or CrCL < 70 mL/min) with previously untreated CLL/SLL. Primary analysis results from the GLOW study demonstrated a significantly improved PFS and greater depth of remission (CR rates and uMRD rates) with ibrutinib plus venetoclax compared with chlorambucil plus obinutuzumab [23].

In European countries, venetoclax has been approved for the treatment of CLL since 2016. It can be administered in combination with obinutuzumab in patients who have not previously been treated or with rituximab in patients who have received at least one previous line of treatment. Single-agent venetoclax can be used in patients with TP53 mutated or 17p deleted who are not eligible for or are resistant to ibrutinib/acalabrutinib or idelalisib. It is also indicated in those patients without these genetic features if they are resistant to chemoimmunotherapy or BTKi [24].

The types of prior first-line therapy, duration of remission, and acquired resistance to treatment are important factors in the selection of treatment for relapsed/refractory CLL. Acalabrutinib, ibrutinib, and venetoclax ± rituximab are approved regimens for the treatment of relapsed/refractory CLL based on the results of phase 3 randomized studies (ASCEND, RESONATE, and MURANO trials, respectively). The PFS benefit compared to the standard chemoimmunotherapy arm was seen across all patient subgroups and particularly those with del(17p) or TP53 mutation. The phase 3 ELEVATE-RR trial demonstrated that acalabrutinib is non-inferior to ibrutinib in terms of PFS and was also associated with a more favorable safety profile in patients with relapsed/refractory del(17p).

The phosphoinositide 3-kinase (PI3K) pathway has been targeted for the treatment of CLL with the development of PI3Ki such as idelalisib or duvelisib. These agents inhibit one or more of the enzymes that are part of the PI3K/AKT/mTOR pathway, responsible for cellular growth and survival. However, due to their toxicity profile, their development in CLL has been hampered and the approval is limited to patients with relapsed disease, and even so, should be administered by physicians with experience in managing their toxicities [15].

In European countries, idelalisib is approved for the treatment of CLL and mainly used in certain patients. It is often administered in combination with a monoclonal antibody (rituximab or ofatumumab), after at least one previous treatment for those harboring TP53 mutation/17p deletion not eligible for other treatments.

It is important to point out that targeted therapies have unique adverse events. Diarrhea, fatigue, arthralgia, infections, cytopenias, bleeding, and cardiovascular toxicities (including atrial fibrillation, ventricular arrhythmias, and hypertension) are the adverse events (AEs) commonly associated with BTKis. Acalabrutinib and zanubrutinib appear to have a more favorable toxicity profile compared to ibrutinib, possibly due to the more selective/specific inhibition of BTK. In the ELEVATE-RR treatment discontinuation due to AEs, it was lower with acalabrutinib (15% vs. 21% for ibrutinib). Atrial fibrillation (9% vs. 16%), hypertension (9% vs. 23%), and bleeding (38% vs. 51%) were less frequent with acalabrutinib compared to ibrutinib. Treatment with acalabrutinib was associated with a higher rate of headache (35% vs. 20% for ibrutinib), with only 2% of patients experiencing grade ≥ 3 headache [14]. Treatment with zanubrutinib was also associated with a substantially lower rate of atrial fibrillation (2.5% vs. 10%) compared to ibrutinib in the ALPINE trial. The benefit and risk of BTKis should be evaluated in patients requiring anti-platelet or anticoagulant therapies. Patients requiring warfarin were excluded from clinical trials evaluating acalabrutinib and ibrutinib, the use of antiaggregants and anticoagulants including warfarin was not restricted in clinical trials evaluating zanubrutinib; however, there were a very small number of patients treated concomitantly. Treatment with idelalisib or duvelisib has been associated with hepatotoxicity (transaminase elevations), severe diarrhea or colitis, pneumonitis, opportunistic infections, and febrile neutropenia. Hepatotoxicity is a major concern in younger patients treated with idelalisib as first-line therapy. Close monitoring of transaminase levels is essential and the concurrent administration of idelalisib or duvelisib with other hepatotoxic drugs should be avoided. The addition of anti-CD20 mAb or chemoimmunotherapy to idelalisib increases the risk of febrile neutropenia. Anti-infective prophylaxis for herpes simplex virus (HSV), pneumocystis jirovecii pneumonia (PJP), and cytomegalovirus (CMV) reactivation are recommended for patients receiving idelalisib or duvelisib [16,25].

Venetoclax can be associated with rapid treatment responses and cause serious and life-threatening tumor lysis syndrome (TLS) in patients with high disease burden. In early phase 1 trials with venetoclax, there were two fatal cases associated with TLS: one in a patient treated with a starting dose higher than the currently recommended 20 mg and the other in a patient whose dose was escalated to 1200 mg. Currently, a target daily dose of 400 mg is recommended in patients with CLL following a 5-week long ramp-up [17]. Consequently, guidelines for the initiation, escalation, and monitoring of treatment with venetoclax have been created and universally implemented together with patients’ risk stratification for TLS [26]. As a result of adherence to these guidelines for the prevention of TLS, clinical trials reported a low incidence (~1.1% to 3.8%) of laboratory-confirmed TLS, with no cases of serious clinical TLS [3,6,7].

**Figure 2:**
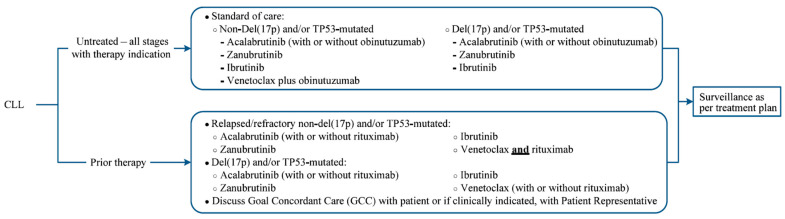
Algorithm detailing treatment options (modified and reproduced with permission from MD Anderson clinical tools, cancer treatment algorithm).

At the present time, the decision to use which therapy for each patient has to be made after considering many factors which include the following: the age of the patient, his/her fitness level, a detailed cardiac history and current level of blood pressure control, prior history of bleeding, ongoing anticoagulation therapy, and a review of medications due to possible drug–drug interactions. Other important elements that should always be assessed before each line of therapy are the presence or absence of genetic mutations such as TP53 or del17p and the acquisition of resistance mutations for patients treated with targeted agents. A detailed chart as shown in Figure 2 summarizes the approach for choosing a therapy depending on the clinical and genetic profiles of the patient.

## 6. Future Directions

Targeted agents are becoming the mainstay of therapy for CLL, with several emerging time-limited combinations able to achieve a high rate of deep complete remissions. Patients in general have tolerated targeted therapies well with side effects that are manageable. It should be considered that resistance to therapy continues to be a relevant problem. Therefore, the rise in genetic mutations leading to resistance has driven the development of newer drugs. The most frequent mechanisms of resistance observed in ibrutinib-treated patients are point mutations: the BTK mutation C481S (a mutation in BTK altering the configuration of the cysteine binding site) and mutations in phospholipase C gamma 2 (PCLg2) [27]. Non-covalent BTKis are a new generation of agents that exert their inhibition of BTK in a manner not influenced by BTK turnover and can inhibit wild-type as well as C481S-mutated BTK [19]. Several non-covalent BTKis are currently under investigation; they include pirtobrutinib, vecabrutinib, nemtabrutinib, and fenebrutinib. Among the non-covalent BTKis, pirtobrutinib has been studied in the phase 2–3 clinical trial BRUIN [28,29,30]. This study enrolled 323 patients with prior use of covalent BTKi, the presence or not of the C481 mutation, and with a large prevalence of high-risk biological features such as 17p (25% of patients) and TP53 mutations (30% of patients). Patients with “triple-refractory” CLL (refractory to covalent BTKi, PI3K inhibitor and venetoclax) had an ORR of 58% which is suggesting clinical activity in this difficult-to-treat population. Acquired resistance to venetoclax can also occur during therapy. Several mechanisms can contribute to this resistance: clonal shifts with the expansion of clones with higher genomic instability and copy number alterations in genes such as NOTCH1, SF3B1, and TP53, point mutations occurring at the BH3-binding pocket of the BCL-2 protein, and increased dependence of the CLL cells on other anti-apoptotic pathway such as MCL-1. The large number of therapeutic options and therapeutic strategies already available or under investigation suggest that in the upcoming years we will continue to see an improvement in survival and quality of life in patients with CLL.

## Figures and Tables

**Table 1:** (**A**) Rai and (**B**) Binet staging systems.

**(A)**		
**Rai Stage**	**Description**	**Modified Risk Status**
0	Lymphocytosis, lymphocytes in blood > 5 × 10^9^/L clonal B cells, and/or >40% lymphocytes in the bone marrow	Low
I	Stage 0 with enlarged lymph node(s)	Intermediate
II	Stage 0–I with splenomegaly, hepatomegaly, or both	Intermediate
III	Stage 0–II with hemoglobin < 11.0 g/dL or hematocrit < 33%	High
IV	Stage 0–III with platelets < 100,000/mm^3^	High
**(B)**		
**Binet Stage**	**Description**	
A	Hemoglobin ≥ 10 g/dL and platelets ≥ 100,000/mm^3^ and <3 enlarged areas	
B	Hemoglobin ≥ 10 g/dL and platelets ≥ 100,000/mm^3^ and ≥3 enlarged areas	
C	Hemoglobin < 10 g/dL and platelets < 100,000/mm^3^ and any number of enlarged areas	

**Table 2:** Genetic prognostic factors for CLL [3,7,8,9,10].

Method of Detection	Prognostic Variable	Risk Category
Interphase cytogenetics (FISH)	Del(17p)	Unfavorable
	Del(11q)	Unfavorable
	+12	Intermediate
	Normal	Intermediate
	Del(13q) (as a sole abnormality)	Favorable
DNA sequencing	TP53	Wild type: Favorable Mutated: unfavorable
	IGHV	>2% mutation: favorable ≤2% mutation: unfavorable
CpG stimulated metaphase karyotype	CK (≥3 unrelated clonal chromosome abnormalities in more than one cell on karyotype)	Unfavorable
Serum Levels	B2M	Elevated: poor prognosis
	LDH	Elevated above normal limit: worse prognosis

## Data Availability

Not applicable.

## References

[1] Chronic Lymphocytic Leukemia—Cancer Stat Facts. https://seer.cancer.gov/statfacts/html/clyl.html.

[2] Tambaro F.P., Wierda W.G. (2020). Tumour Lysis Syndrome in Patients with Chronic Lymphocytic Leukaemia Treated with BCL-2 Inhibitors: Risk Factors, Prophylaxis, and Treatment Recommendations. Lancet Haematol..

[3] Hallek M., Al-Sawaf O. (2021). Chronic Lymphocytic Leukemia: 2022 Update on Diagnostic and Therapeutic Procedures. Am. J. Hematol..

[4] Burger J.A., Barr P.M., Robak T., Owen C., Ghia P., Tedeschi A., Bairey O., Hillmen P., Coutre S.E., Devereux S. (2019). Long-Term Efficacy and Safety of First-Line Ibrutinib Treatment for Patients with CLL/SLL: 5 Years of Follow-up from the Phase 3 RESONATE-2 Study. Leukemia.

[5] Wang E., Mi X., Thompson M.C., Montoya S., Notti R.Q., Afaghani J., Durham B.H., Penson A., Witkowski M.T., Lu S.X. (2022). Mechanisms of Resistance to Noncovalent Bruton’s Tyrosine Kinase Inhibitors. N. Engl. J. Med..

[6] (2023). NCCN Clinical Practice Guidelines in Oncology Version 1. https://www.nccn.org/guidelines/guidelines-detail?category=1&id=1478.

[7] Montserrat E., Marques-Pereira J.P., Rozman C., Ballesta A.M., Aguilar J.L., Elena M. (1982). Serum Beta-2 Microglobulin in Chronic Lymphocytic Leukaemia. Clin. Lab. Haematol..

[8] Hallek M., Wanders L., Ostwald M., Busch R., Senekowitsch R., Stern S., Schick H.-D., Kuhn-Hallek I., Emmerich B. (1996). Serum β_2_-Microglobulin and Serum Thymidine Kinase Are Independent Predictors of Progression-Free Survival in Chronic Lymphocytic Leukemia and Immunocytoma. Leuk. Lymphoma.

[9] Autore F., Strati P., Innocenti I., Corrente F., Trentin L., Cortelezzi A., Visco C., Coscia M., Cuneo A., Gozzetti A. (2019). Elevated Lactate Dehydrogenase Has Prognostic Relevance in Treatment-Naïve Patients Affected by Chronic Lymphocytic Leukemia with Trisomy 12. Cancers.

[10] Barr P.M., Owen C., Robak T., Tedeschi A., Bairey O., Burger J.A., Hillmen P., Coutre S.E., Dearden C., Grosicki S. (2022). Up to 8-Year Follow-up from RESONATE-2: First-Line Ibrutinib Treatment for Patients with Chronic Lymphocytic Leukemia. Blood Adv..

[11] Hallek M., Cheson B.D., Catovsky D., Caligaris-Cappio F., Dighiero G., Döhner H., Hillmen P., Keating M., Montserrat E., Chiorazzi N. (2018). IwCLL Guidelines for Diagnosis, Indications for Treatment, Response Assessment, and Supportive Management of CLL. Blood.

[12] Early Intervention in Asymptomatic Chronic Lymphocytic Leukemia—Hematology & Oncology. https://www.hematologyandoncology.net/archives/february-2021/early-intervention-in-asymptomatic-chronic-lymphocytic-leukemia/.

[13] Eichhorst B., Robak T., Montserrat E., Ghia P., Niemann C.U., Kater A.P., Gregor M., Cymbalista F., Buske C., Hillmen P. (2021). Chronic Lymphocytic Leukaemia: ESMO Clinical Practice Guidelines for diagnosis, treatment and follow-up. Ann. Oncol..

[14] Desikan S.P., Venugopal S., Ferrajoli A. (2022). BTK inhibitor selection for chronic lymphocytic leukemia: Which drug for which patient?. Expert Rev. Hematol..

[15] Hus I., Puła B., Robak T. (2022). PI3K inhibitors for the treatment of chronic lymphocytic leukemia: Current status and future perspectives. Cancers.

[16] Ahn I.E., Tian X., Wiestner A. (2020). Ibrutinib for chronic lymphocytic leukemia with *tp53* alterations. N. Engl. J. Med..

[17] Fakhri B., Andreadis C. (2021). The role of acalabrutinib in adults with chronic lymphocytic leukemia. Ther. Adv. Hematol..

[18] Delgado J., Josephson F., Camarero J., Garcia-Ochoa B., Lopez-Anglada L., Prieto-Fernandez C., Hennik P.B., Papadouli I., Gisselbrecht C., Enzmann H. (2021). Ema Review of acalabrutinib for the treatment of adult patients with chronic lymphocytic leukemia. Oncologist.

[19] Lewis K.L., Cheah C.Y. (2021). Non-Covalent BTK Inhibitors-the New BTKids on the Block for B-Cell Malignancies. J. Pers. Med..

[20] Brown J.R., Eichhorst B., Hillmen P., Jurczak W., Kaźmierczak M., Lamanna N., O’Brien S.M., Tam C.S., Qiu L., Zhou K. (2023). Zanubrutinib or Ibrutinib in relapsed or refractory chronic lymphocytic leukemia. N. Engl. J. Med..

[21] Al-Sawaf O., Zhang C., Tandon M., Sinha A., Fink A.-M., Robrecht S., Samoylova O., Liberati A.M., Pinilla-Ibarz J., Opat S. (2020). Venetoclax plus obinutuzumab versus Chlorambucil Plus obinutuzumab for previously untreated chronic Lymphocytic Leukaemia (cll14): Follow-up results from a multicentre, open-label, randomised, phase 3 trial. Lancet Oncol..

[22] Tam C.S., Allan J.N., Siddiqi T., Kipps T.J., Jacobs R., Opat S., Barr P.M., Tedeschi A., Trentin L., Bannerji R. (2022). Fixed-Duration Ibrutinib plus Venetoclax for First-Line Treatment of CLL: Primary Analysis of the CAPTIVATE FD Cohort. Blood.

[23] Kater A.P., Owen C., Moreno C., Follows G., Munir T., Levin M.-D., Benjamini O., Janssens A., Osterborg A., Robak T. (2022). Fixed-duration ibrutinib-venetoclax in patients with chronic lymphocytic leukemia and Comorbidities. NEJM Evid..

[24] Waggoner M., Katsetos J., Thomas E., Galinsky I., Fox H. (2022). Practical management of the Venetoclax-treated patient in chronic lymphocytic leukemia and acute myeloid leukemia. J. Adv. Pract. Oncol..

[25] Kay N.E., Hampel P.J., Van Dyke D.L., Parikh S.A. (2022). CLL Update 2022: A Continuing Evolution in Care. Blood Rev..

[26] Gribben J.G. (2019). Practical Management of Tumour Lysis Syndrome in Venetoclax-Treated Patients with Chronic Lymphocytic Leukaemia. Br. J. Haematol..

[27] Burger J.A. (2021). Integrating new therapies for chronic lymphocytic leukemia. Cancer J..

[28] Michot J.-M., Ribrag V. (2021). Pirtobrutinib Shows Evidence to Inaugurate a Third Generation of BTK Inhibitors. Lancet.

[29] Reiff S.D., Muhowski E.M., Guinn D., Lehman A., Fabian C.A., Cheney C., Mantel R., Smith L., Johnson A.J., Young W.B. (2018). Noncovalent Inhibition of C481S Bruton Tyrosine Kinase by GDC-0853: A New Treatment Strategy for Ibrutinib-Resistant CLL. Blood.

[30] NEJM Journal Watch: Summaries of and Commentary on Original Medical and Scientific Articles from Key Medical Journals. https://www.jwatch.org/na53334/2021/05/20/third-generation-bruton-tyrosine-kinase-inhibitor.

